# Single-incision laparoscopic surgery (SILS) for the treatment of ileocolonic Crohn’s disease: a propensity score–matched analysis

**DOI:** 10.1007/s00384-020-03821-6

**Published:** 2020-12-23

**Authors:** Valerio Celentano, Gianluca Pellino, Matteo Rottoli, Francesco Colombo, Gianluca Sampietro, Antonino Spinelli, Francesco Selvaggi, SICCR Current status of Crohn’s disease surgery collaborative, Valerio Celentano, Gianluca Pellino, Matteo Rottoli, Gilberto Poggioli, Giuseppe Sica, Mariano Cesare Giglio, Michela Campanelli, Claudio Coco, Gianluca Rizzo, Francesco Sionne, Francesco Colombo, Gianluca Sampietro, Giulia Lamperti, Diego Foschi, Ferdinando Ficari, Ludovica Vacca, Marta Cricchio, Francesco Giudici, Lucio Selvaggi, Guido Sciaudone, Roberto Peltrini, Andrea Manfreda, Luigi Bucci, Raffaele Galleano, Omar Ghazouani, Luigi Zorcolo, Simona Deidda, Angelo Restivo, Andrea Braini, Francesca Di Candido, Matteo Sacchi, Michele Carvello, Stefania Martorana, Giovanni Bordignon, Imerio Angriman, Angela Variola, Mirko Di Ruscio, Giuliano Barugola, Andrea Geccherle, Francesca Paola Tropeano, Gaetano Luglio, Marta Tanzanu, Diego Sasia, Marco Migliore, Maria Carmela Giuffrida, Enrico Marrano, Gianluigi Moretto, Harmony Impellizzeri, Gaetano Gallo, Giuseppina Vescio, Giuseppe Sammarco, Giovanni Terrosu, Giacomo Calini, Andrea Bondurri, Anna Maffioli, Gloria Zaffaroni, Andrea Resegotti, Massimiliano Mistrangelo, Marco Ettore Allaix, Fiorenzo Botti, Matteo Prati, Luigi Boni, Serena Perotti, Michela Mineccia, Antonio Giuliani, Lucia Romano, Giorgio Maria Paolo Graziano, Luigi Pugliese, Andrea Pietrabissa, GianGaetano Delaini, Antonino Spinelli, Francesco Selvaggi

**Affiliations:** 1 Portsmouth Hospitals NHS Trust, Portsmouth, UK; 2 University of Portsmouth, Portsmouth, UK; 3 Department of Surgery and Cancer, Imperial College, London, UK; 4 Department of Advanced Medical and Surgical Sciences, Universita’ degli Studi della Campania Luigi Vanvitelli, Naples, Italy; 5 Colorectal Surgery, Vall d’Hebron University Hospital, Barcelona, Spain; 6 Surgery of the Alimentary Tract, Sant’Orsola Hospital, Department of Medical and Surgical Sciences, Alma Mater Studiorum University of Bologna, Bologna, Italy; 7 General Surgery Unit, Department of Biomedical and Clinical Sciences “L. Sacco”, University of Milan, AAST Fatebenefratelli Sacco, Milan, Italy; 8 Division of General and HPB Surgery, Department of Surgery, ASST Rhodense - Rho Memorial Hospital, 20017 Rho, Milan, Italy; 9 Division of Colon and Rectal Surgery, Humanitas Clinical and Research Center IRCCS, Via Manzoni 56, 20089 Rozzano, Milan, Italy; 10 Department of Biomedical Sciences, Humanitas University, Via Rita Levi Montalcini 4, 20090 Pieve Emanuele, Milan, Italy

**Keywords:** Single-incision laparoscopic surgery, Inflammatory Bowel Disease, Crohn’s disease

## Abstract

**Introduction:**

Single-incision laparoscopic surgery (SILS) aims to minimize the surgical access trauma by reducing the number of abdominal incisions to a single site, potentially offering better cosmetic results and decreased postoperative pain. In this study, we compare the results of SILS ileocolic resection for Crohn’s disease (CD) to conventional laparoscopy and open surgery using a propensity score–matched analysis in a retrospective national multicentre study.

**Methods:**

All consecutive patients undergoing elective SILS ileocaecal or redo ileocolic resection for primary and recurrent CD from 1 June 2018 to 31 May 2019 were included. Patients were matched 1:1:1 with laparoscopy and open surgery according to perianal disease, recurrent disease, penetrating phenotype of CD, history of previous abdominal surgery, preoperative medical treatment with steroids and anti-TNF. Postoperative morbidity within 30 days of surgery was the primary endpoint.

**Results:**

Fifty-eight patients were included in each group, for a total of 174 patients. The conversion rate for SILS and laparoscopy was 10.3% and 12%, respectively, with no difference in the incidence of postoperative complications (13.8% and 12%, *p* = 0.77), whilst open surgery demonstrated a worse morbidity profile, with a complication rate of 25.9% (*p* < 0.0001). Median length of hospital stay following SILS ileocolic resection was 5 days, significantly shorter compared to 7 days for laparoscopy and 9 for open surgery (*p* < 0.0001).

**Conclusions:**

SILS ileocolonic resection for CD demonstrated a comparable morbidity profile compared to laparoscopy in selected patients, with a reduced length of postoperative hospital stay.

**Supplementary Information:**

The online version contains supplementary material available at 10.1007/s00384-020-03821-6.

## Introduction

Patients with Crohn’s disease (CD) are at high risk of recurrence and reoperation following ileocaecal resection. Minimally invasive surgery techniques offer several advantages including reduced postoperative adhesion formation which may facilitate redo surgery [1].

Single-incision laparoscopic surgery (SILS) aims to minimize the surgical access trauma by reducing the number of abdominal incisions to a single incision site, potentially offering better cosmetic results [2] and decreased postoperative pain [3]. However, this procedure is relatively recent, and some variability exists in published studies [2], advocating for more data on the topic. In this study, we compare the results of SILS ileocaecal resection to conventional laparoscopy and open surgery using a propensity score–matched analysis extracted from the national, multicentre study “Current status of Crohn’s disease surgery”, promoted by the Italian Society of Colorectal Surgery (SICCR).

## Methods

### Study settings

The Italian Society of Colorectal Surgery (SICCR) promoted the snapshot study “Current status of Crohn’s disease surgery”, which is a retrospective, multicentre, observational study [4] developed according to the STROBE checklist [5]. Ethical approval was obtained from the promoting centres and every participating centre had a named Principal Investigator, liaising with the local ethics committee. Obtaining informed consent from the patients was deemed not necessary by the Ethics Committees in view of the retrospective nature of the study.

### Eligibility criteria

All consecutive patients (aged 16 or older) undergoing elective SILS ileocaecal or redo ileocolic resection for primary and recurrent CD from 1 June 2018 to 31 May 2019 were included. Patients undergoing proctocolectomy, proctectomy or segmental colectomy were excluded from this study. SILS was defined as surgery performed with multiple instruments or trocars introduced via a single incision site, with the choice of commonly available dedicated SILS ports [6] or use of surgical glove-port [7] left to the individual surgeons’ preference. Procedures performed with hand-assisted surgery were excluded. If additional trocars were inserted during the SILS procedure, it was considered converted to conventional laparoscopy with intention to treat analysis.

### Propensity score–matched analysis

To compare the results of SILS ileocaecal resections, patients were matched 1:1:1 with laparoscopy and open surgery according to presence of perianal disease, recurrent disease, penetrating phenotype of CD, history of previous abdominal surgery, preoperative medical treatment with steroids and anti-TNF.

### Study objectives

Postoperative morbidity within 30 days of surgery was the primary endpoint. Postoperative length of hospital stay (LOS) and stoma rate were the secondary outcome measures.

### Statistical analysis

Categorical variables are presented as frequency and percentages, and were compared using the chi-square test or Fisher’s exact test, as appropriate. Continuous variables are presented as mean (±standard deviation) or median (range) according to their distribution, and were compared with the use of Student’s *t* test or the Mann-Whitney *U* test in case of normal or skewed distribution, respectively. All reported *p* values were two-tailed, and *p* values of less than 0.05 were considered to be statistically significant. Statistical analysis was performed by using IBM SPSS Statistics for Windows, version 25.0 (IBM Corp., Armonk, NY, USA).

## Results

Fifty-eight patients were included in each group, for a total of 174 patients. Only 5 patients (8.6%) in the SILS group had a penetrating phenotype of disease (Table 1).

**Table 1:** Baseline characteristics of the included patients

	SILS (*n* = 58)	Laparoscopy (*n* = 58)	Open (*n* = 58)	*p* value
Age	43 (20−77)	42 (24−79)	48 (25−85)	ns
BMI	21.2 (17.3−32.6)	23 (15−31)	20.5 (17−29)	ns
Previous surgery	18 (31%)	18 (31%)	18 (31%)	ns
Primary/recurrent	43/15	43/15	43/15	ns
Penetrating disease	5 (8.6%)	5 (8.6%)	5 (8.6%)	ns
Perianal disease	11 (19%)	11 (19%)	11 (19%)	ns
Preop steroids	10 (17.2%)	10 (17.2%)	10 (17.2%)	ns
Preop anti-TNF	9 (15.5%)	9 (15.5%)	9 (15.5%)	ns

*SILS* single-incision laparoscopic surgery, *n* number, *BMI* body mass index, *TNF* tumour necrosis factor, *ns* not statistically significant

The conversion rate for SILS and laparoscopy was 10.3% and 12%, respectively, with no difference in the incidence of postoperative complications (13.8% and 12%, *p* = 0.77), whilst open surgery demonstrated a worse morbidity profile, with a complication rate of 25.9% (*p* < 0.0001). Median LOS following SILS ileocolic resection was 5 days, significantly shorter compared to 7 days for laparoscopy and 9 for open surgery (*p* < 0.0001) (Table 2).

**Table 2:** Surgical outcomes

	SILS (*n* = 58)	Laparoscopy (*n* = 58)	Open (*n* = 58)	*p* value
Conversion	6 (10.3%)	7 (12%)	N/A	0.77
Ileostomy	8 (13.8%)	4 (6.9%)	8 (13.8%)	0.22
LOS	5 (3−20)	7 (4−15)	9 (4−66)	< 0.0001
Complications	8 (13.8%)	7 (12%)	15 (25.9%)	< 0.0001
Details of complications	3 intra-abdominal collections treated with antibiotics, 2 chest infections 1 bleeding requiring transfusions 2 wound infections	1 anastomotic leak 2 urinary infections 2 small bowel obstructions treated conservatively 2 bleeding requiring transfusions	2 chest infections 1 DVT 4 anastomotic leaks 1 small bowel injury 1 CVC sepsis 3 wound infections 1 small bowel obstruction treated conservatively	
Readmissions	0	0	1 (1.7%)	0.32
Reoperations	0	1 (1.7%) for anastomotic leak	4 (6.9%) 3 for anastomotic leak 1 for small bowel resection	0.04

*SILS* single-incision laparoscopic surgery, *n* number, *LOS* length of hospital stay, *N/A* not applicable, *DVT* deep vein thrombosis, *CVC* central venous catheter

## Discussion

Our study found that patients undergoing SILS for ileocolonic CD have a similar postoperative morbidity compared to conventional laparoscopic and a shorted LOS. Open surgery demonstrated a twofold increase in postoperative complications and a LOS of approximately 4 and 2 days longer compared to SILS and conventional laparoscopy, respectively.

Laparoscopy is clearly associated with several short-term benefits in adult and paediatric patients with CD, including shorter time to restoration of bowel function, reduced wound complications and LOS, and better cosmesis [8, 9]. SILS represents a further step forward towards minimally invasive surgery in CD [10], but several aspects related to its feasibility remain debated, particularly in view of specific technical challenges posed by CD such as the presence of adhesions and internal fistulae, the thickened vascular mesentery and the subtle nature of some multisite small bowel strictures [11].

It is important to consider that only few studies have been published to date concerning SILS in CD with reported variability in several aspects of the procedure. A review of 11 studies, including 369 patients, identified differences in the indication for surgery and the type of port and camera used [2]. Several reports included patients in whom additional trocars were placed or a hand-assisted procedure has been described. Postoperative morbidity ranges between 4.1 and 18.8% following SILS for CD, with reoperation rates not exceeding 6%, and an average LOS of 4–11 days [2]. A multicentre study compared the outcomes of SILS with that of conventional laparoscopy for ileocaecal CD, and found that SILS was associated with reduced postoperative pain and analgesia requirements [3]. However, SILS might be less suitable for complex CD operations as for example patients requiring additional procedures such as multiple bowel resections or strictureplasties.

The findings of our study confirm that SILS is currently applied in a selected group of CD patients, as we found a rate of penetrating disease of only 8.6%, with a relatively high rate of ileostomy formation (13.8%) in this cohort of patients undergoing elective surgery. The number of patients requiring an ileostomy in the SILS group was doubled compared to laparoscopy, but it did not reach statistical significance, likely due to the small sample size. The ileostomy rate is an important key performance indicator in CD surgery, and must be evaluated in prospectively maintained databases.

We must acknowledge as a limitation of our study the risk of information and recall bias, due to the retrospective design and also the self-reported nature of our data from many different centres. It is possible that patients undergoing conventional laparoscopy or open surgery had a more technically challenging procedure, or reached surgery in a more deconditioned status compared to the SILS group. However, the propensity score–matched analysis allowed comparison of CD patients with similar characteristics undergoing ileocolonic resection in the three different groups, obtaining meaningful results to support the use of SILS in selected patients. Data on Patient-Reported Outcome Measures (PROMs) was not included despite it may provide an essential role when deciding between surgical approaches in patients with CD. We previously demonstrated that the anastomotic technique or configuration does not affect CD postoperative morbidity as a single causative factor [12], and similarly it is important to highlight the need for multidisciplinary-led decision-making to offer the most appropriate surgical approach tailored to the specific characteristics of the individual patient, moving the focus from a single causative factor (i.e. anastomotic technique, preoperative treatment) to a multifactorial approach.

## Conclusions

SILS ileocolonic resection for CD demonstrated a comparable morbidity profile compared to laparoscopy in selected patients, with a reduced length of postoperative hospital stay.

## Supplementary Information

ESM 1 (DOCX 19.2 kb)

ESM 2 (DOCX 14.5 kb)
